# Supplementary figures and images for: A generic method for PLC control system design based on Digital Twin
Source: PLoS One. 2025 Jul 3;20(7):e0327592. doi: 10.1371/journal.pone.0327592 (PMC12225803; doi:10.1371/journal.pone.0327592)

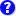

Supplement: S1 File — (ZIP) [file pone.0327592.s001.zip › Original program/elevator20231127/Logs/ICO_PE_InfoActionRequest.png]

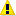

Supplement: S1 File — (ZIP) [file pone.0327592.s001.zip › Original program/elevator20231127/Logs/ICO_PE_InfoDecisionCritical.png]

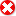

Supplement: S1 File — (ZIP) [file pone.0327592.s001.zip › Original program/elevator20231127/Logs/ICO_PE_InfoError.png]

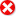

Supplement: S1 File — (ZIP) [file pone.0327592.s001.zip › Original program/elevator20231127/Logs/ICO_PE_InfoErrorCritical.png]

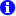

Supplement: S1 File — (ZIP) [file pone.0327592.s001.zip › Original program/elevator20231127/Logs/ICO_PE_InfoInformation.png]

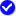

Supplement: S1 File — (ZIP) [file pone.0327592.s001.zip › Original program/elevator20231127/Logs/ICO_PE_InfoSuccess.png]
