# Supplementary material for: A generic method for PLC control system design based on Digital Twin
Source: PLoS One. 2025 Jul 3;20(7):e0327592. doi: 10.1371/journal.pone.0327592 (PMC12225803; doi:10.1371/journal.pone.0327592)
Supplement: Attachment — Submitted filename: Response to Reviewers_20250121.docx [file pone.0327592.s003.docx]

Dear Editors and Reviewers:

Thank you very much for your comments and professional advice. These comments are of great help in enhancing the academic rigor of our manuscript, and they also have guiding significance for us to carry out relevant research work and write academic papers in the future. We would like to express our sincere gratitude again! According to your advice and requirements, we have revised and improved the manuscript. We added the missing content and necessary explanations, conducted quantitative analysis of the experimental data, improved annotations of several figures, and deleted unnecessary paragraphs. We greatly appreciate your re-review of the revised manuscript. We look forward to further improvements of our work. The following are the specific details of the manuscript revisions based on the reviewers' comments:

**Reviewer #1:**

**Comment 1:** *Section 0: why should digital twins be integrated into electrical control systems? The challenges to be addressed by this work should be clarified.*

**Response:**

We are extremely grateful to you for pointing out the issue. The previous manuscript did not clearly explain this issue. Now, we have added relevant content to the introduction section, clarifying that a complete Digital Twin is not merely the Digital Twin of production equipment, but should also include the Digital Twin of the corresponding electrical control system. Moreover, we have explained the important role of the Digital Twin of the electrical control system in the design, commissioning, and process improvement of the production line.

**Comment 2:** *Section 1.2: the differences between virtual commissioning and traditional commissioning methods are not summarized clearly. What are the advantages and disadvantages of virtual commissioning and traditional commissioning?*

**Response:**

We are extremely grateful to you for pointing out the issue. The previous manuscript did not have a clear summary of the differences between virtual commissioning and traditional commissioning methods, and the respective advantages and disadvantages of these two methods were not expounded clearly. Now, we have added relevant content to clearly summarize the differences between them and their respective advantages and disadvantages.

**Comment 3:** *Section 1.3: the control system design process is a reference to someone else's work and the innovation of this work should be clarified.*

**Response:**

We are extremely grateful to you for pointing out the issue. The previous manuscript did not clearly summarize the innovativeness of this research work. Now, we have added relevant content to clarify that the uniqueness of this research lies in the following: From the perspective of the end-user of Digital Twin, for an electrical control engineer who doesn't have in - depth knowledge of the relevant technologies for establishing Digital Twin, what kind of path should be followed to achieve the design of an electrical control system based on Digital Twin.

**Comment 4:** *Section 3.3: the designed software interface should clearly identify the different areas and describe their functions in English, rather than merely presenting a screenshot of the interface.*

**Response:**

We are extremely grateful to you for pointing out the issues with the figures in Section 3.3. We have revised the relevant figures.

- The interface related to Figure 7 has already appeared in Figure 4 earlier. Therefore, we have annotated the functions of each part of the software interface in Figure 4. Meanwhile, we have clearly annotated the significance of the newly added content in Figure 7.

- In Figure 8, we add annotations for the relevant content.

- To be more in line with the description of the relevant content, we have replaced Figure 9 and added annotations.

- Also, to better match the relevant description, we have revised Figure 10 by adding two figures. The comparison between the figures can more clearly demonstrate how the data exchange settings are implemented.

- In Figure 12, we add annotations for the relevant content.

**Comment 5:** *Section 3.5: the vertical axis in Figure 13 lacks a clear explanation. The meaning of this coordinate should be clarified, and the software results should be quantitatively presented.*

**Response:**

We are extremely grateful to you for pointing out the issue. When referring to Figure 13 in the manuscript, we have added relevant content. Specifically, we have explained the meanings of the ordinate and abscissa, changed the display method to more prominently highlight the scores of each item. Moreover, we have introduced the main influencing factors of the scores in the four virtual commissioning processes, as well as the improvements made to the virtual control system based on the feedback results. This clarifies the significance of the presented data.

**Comment 6:** *Section 4: the methodology employed in this work is not adequately addressed, and the experimental results are not quantified. These aspects should be explicitly included to provide a more comprehensive conclusion.*

**Response:**

We are extremely grateful to you for pointing out the issue. We have added relevant content in Sections 3.5 and 4. First, in Section 3.5, we added explanations and analyses of the experimental data. Then, in Section 4, we added the summary and elaboration of the research work and methods involved in the manuscript, hoping to provide a more comprehensive conclusion.

**Reviewer #2:**

**Comment 1:** *The number of references cited in this manuscript is relatively limited, which to some extent constrains the theoretical depth and breadth of the article. It is recommended that the author broaden the scope of literature searches in subsequent research and writing, incorporating citations of classic literature and the latest research findings in the relevant field.*

**Response:**

We sincerely appreciate your valuable suggestions. As electrical control engineers mainly engaged in technical application work, our exploration and research on the depth and breadth of the theory of our article are indeed lacking. We will definitely strengthen our efforts in this regard during our subsequent research and writing. We will search for and study more classic literatures and the latest research findings. We believe these efforts will be of great help in improving our work capabilities and research levels. Thank you again for your precious suggestions!

**Comment 2:** *I have observed instances of nearly identical paragraphs. Specifically, within the 2.2 Application of DT maturity analysis section, the paragraphs titled "DT Model dimension" and "Dimension of DT model," as well as the opening paragraphs of the "In summary" section, exhibit a high degree of similarity, seemingly reiterating the same information. Avoiding content repetition is crucial in academic writing, and it is advisable for the author to revise and adjust these paragraphs accordingly.*

**Response:**

We are extremely grateful to you for pointing out the issue. It was indeed due to our oversight that these repetitive contents occurred. Your review has helped us avoid such elementary mistakes. We have deleted the repetitive contents. Thank you again!

**Comment 3:** *The conclusion analysis constitutes a vital component of the article, yet the conclusion analysis and system improvement section (3.5 Result analysis and system improvement) appears somewhat underdeveloped, failing to fully explore the significance and potential implications of the research findings. For instance, based on the scoring results, what specific issues exist within the control system during elevator operation, and what corresponding optimization methods are proposed?*

We are extremely grateful to you for pointing out the issue. Regarding this matter, when referring to Figure 13 in the manuscript, we specifically introduced the main influencing factors of the scores in the four virtual commissioning processes, as well as the improvements made to the virtual control system based on the feedback results. This demonstrates the assistance of the virtual commissioning process in enhancing the virtual control system.

**Comment 4:** *The description of Fig. 13 lacks clarity, and there are missing annotations on the graphic, such as the units for the numerical values on the vertical axis, which may confuse readers. It is recommended that the author provide a more detailed description of the chart, including explanations of the horizontal and vertical axes, as well as the legends.*

**Response:**

We are extremely grateful to you for pointing out the issue. When referring to Figure 13 in the manuscript, we have added relevant content. Specifically, we have explained the meanings of the ordinate and abscissa, changed the display method to more prominently highlight the scores of each item. Moreover, we have introduced the main influencing factors of the scores in the four virtual commissioning processes, as well as the improvements made to the virtual control system based on the feedback results. This clarifies the significance of the presented data.

Thank you very much for your attention and time. Look forward to hearing from you.

Yours sincerely

Hao Shi

Corresponding Author

Department of Electrical Engineering and Automation，Shanxi Institute of Technology，Yangquan，Shanxi, China
