# Supplementary material for: A generic method for PLC control system design based on Digital Twin
Source: PLoS One. 2025 Jul 3;20(7):e0327592. doi: 10.1371/journal.pone.0327592 (PMC12225803; doi:10.1371/journal.pone.0327592)
Supplement: Attachment — Submitted filename: Response to Reviewers_20250410.docx [file pone.0327592.s004.docx]

Dear Editors and Reviewers:

Thank you very much for your comments and professional advice. These comments are of great help in enhancing the academic rigor of our manuscript, and they also have guiding significance for us to carry out relevant research work and write academic papers in the future. We would like to express our sincere gratitude again! According to your advice and requirements, we have meticulously revised the manuscript. We highlighted the innovation of this research in the abstract, restructured the conclusion section, and added a description of the potential limitations. We avoided absolute or overly assertive statements, revised informal expressions to formal terminologies, simplified verbose sentences, corrected grammatical errors, and ensured consistent and appropriate tense usage. Additionally, we revised the manuscript title to more directly reflect the core research focus. We revised the text in the figures to align with the manuscript's content. We greatly appreciate your re-review of the revised manuscript. We look forward to further improvements in our work. The following are the specific details of the manuscript revisions based on the reviewers' comments:

**Reviewer #1:**

**Comment:** *The comments have been addressed in the revised manuscript. It is suggested to be accepted for publication.*

**Response:**

We sincerely appreciate your thorough re-review of the manuscript. It is both an honor and a privilege to receive your recognition of our work.

**Reviewer #2:**

**Comment 1:** *The abstract does not clearly state the innovation of this study. It is suggested to supplement the explanation of the innovative aspects of this study.*

**Response:**

We sincerely appreciate your valuable suggestions. We have highlighted the innovation of this study in the abstract. Specifically, we emphasize that this study explores a method for designing Digital Twin (DT)-based electrical control systems from the perspective of an electrical control engineer with limited understanding of DT design and development technologies. This approach addresses the implementation challenges faced by practitioners with limited knowledge of DT technologies. Thank you again for your precious suggestions!

**Comment 2:** *The language is repetitive and verbose. It is suggested to avoid absolute and colloquial expressions, unify the tenses, and make the language more concise.*

**Response:**

We are extremely grateful to you for pointing out the issue. Due to our lack of English writing experience, the previous manuscript had issues in language and tenses. Your review has helped us avoid such basic errors. To address these, we reviewed the publisher’s guidelines and extensively analyzed published academic literature to enhance our understanding of tense conventions and language norms. The revisions were guided by the following principles:

- Tense: Simple present tense is applied to describe the research background, objectives, and discussion of results. Simple past tense is used to detail experimental procedures and outcomes.
- Language: Avoidance of absolute or overly assertive statements. Simplification of verbose sentences and correction of grammatical errors. Replacement of informal expressions with formal terminology.

We have thoroughly revised the entire manuscript, including the title, to enhance its readability and compliance with academic standards. Detailed revisions are highlighted in the “Revised Manuscript with Track Changes” file. The text highlighted in red indicates modifications or additions, while the text highlighted in red with strikethroughs indicates the deleted parts. Thank you again for your review!

**Comment 3:** *The conclusion section mixes the research summary, practical verification, and future prospects in a way that lacks clear hierarchical structure. It is recommended to clearly separate these three parts and add a description of the limitations for the future, which will facilitate the reading and research of the readers.*

**Response:**

We are extremely grateful to you for pointing out the issue. We have restructured the conclusion section into three subsections: Research Summary, Practical Validation, and Future Prospects. Additionally, we added a dedicated discussion on the potential limitations of the research content when applied in future contexts. These revisions are intended to facilitate the reading and research of the readers.

**Reviewer #3:**

**Comment:** *However, there are occasional grammatical errors and awkward phrasings that could benefit from minor editorial improvements.*

**Response:**

We are extremely grateful to you for pointing out the issue. Due to our lack of English writing experience, the previous manuscript had issues in language and tenses. Your review has helped us avoid such basic errors. To address these, we reviewed the publisher’s guidelines and extensively analyzed published academic literature to enhance our understanding of tense conventions and language norms. The revisions were guided by the following principles:

- Tense: Simple present tense is applied to describe the research background, objectives, and discussion of results. Simple past tense is used to detail experimental procedures and outcomes.
- Language: Avoidance of absolute or overly assertive statements. Simplification of verbose sentences and correction of grammatical errors. Replacement of informal expressions with formal terminology.

We have thoroughly revised the entire manuscript, including the title, to enhance its readability and compliance with academic standards. Detailed revisions are highlighted in the “Revised Manuscript with Track Changes” file. The text highlighted in red indicates modifications or additions, while the text highlighted in red with strikethroughs indicates the deleted parts. Thank you again for your review!

Thank you very much for your attention and time. Look forward to hearing from you.

Yours sincerely

Hao Shi

Corresponding Author

Department of Electrical Engineering and Automation, Shanxi Institute of Technology, Yangquan，Shanxi, China
